# Unwanted souvenirs—import routes and pathogen detection of the non-endemic tick *Rhipicephalus sanguineus* s.l. in Germany

**DOI:** 10.1007/s10493-025-01010-0

**Published:** 2025-03-11

**Authors:** K. Fachet-Lehmann, A. Lindau, U. Mackenstedt

**Affiliations:** Department of Parasitology, University of Hohenheim, Stuttgart, Germany

**Keywords:** *Rhipicephalus sanguineus* s.l., Tick introduction, Pathogen detection, Molecular identification, *Rickettsia massiliae*, Germany

## Abstract

Ticks of the Genus *Rhipicephalus* occur worldwide. Especially members of *Rh. sanguineus* s.l. are primarily associated with dogs. As unwanted souvenirs, they are introduced by dogs into non-endemic areas such as Germany, where they can establish and reproduce indoors*.* A citizen-science study was conducted between 2019 and 2024, asking interested citizens to report tick infestations and send in travel related tick findings which were associated with dogs and were focused on *Rhipicephalus* species. Tick species were identified using the 16S rRNA gene and tested for pathogens associated with the genus *Rhipicephalus*. In addition, each tick introduction was considered as a case and categorized and analyzed individually. During the study period, 44 cases were reported. In 17 cases tick specimens were identified as *Rh. sanguineus* s.s., *Rh. rutilus*, *Rh. linnaei, Rh. turanicus*, and *Rh. haemaphysaloides* which were imported from other European countries and Sri Lanka. Neither *Hepatozoon canis*, *Ehrlichia canis* nor *Babesia vogeli* were detected in any of the 780 received specimens. In contrast, *Rickettsia* spp. was detected in 50 specimens from six independent cases, with *Rickettsia massiliae* being identified in 44 specimens. The import of dogs from abroad as well as travel with dogs lacking appropriate tick prophylaxis are responsible for more than 80% of cases. This Project highlights the risk of introductions of non-endemic tick species. Also, professionals such as veterinarians, animal welfare organizations and pest controllers need to be made aware of the possible introduction of *Rhipicephalus* spp. to ensure early recognition and rapid elimination of the ticks.

**Supplementary Information:**

The online version contains supplementary material available at 10.1007/s10493-025-01010-0.

## Introduction

*Rhipicephalus* species are very resilient hard ticks with an adaptation to high temperatures and low humidity which enables them to survive in nearly every part of the world (Walker et al. 2000; Tian et al. 2001; Gray et al. 2013). *Rhipicephalus sanguineus* s.l., as a member of the *Rhipicephalus sanguineus* group, is distributed across the world and is common in urban areas (Camicas et al. 1998; Walker et al. 2000; Szabova et al. 2007; Dantas-Torres 2010; Gray et al. 2013; Hekimoğlu et al. 2016; Hekimoğlu 2024). Members of *Rh. sanguineus* s.l. differ in behaviour, adaptions to climatic conditions, distribution, and vector capacity. They tolerate average annual temperatures of between 10 °C and 30 °C (Nava et al. 2018; Šlapeta et al. 2021) and can endure very low humidity (< 40%) (Tian et al. 2022). Genetic analyses and a morphological redescription in 2018 have redefined the neotype of *Rh. sanguineus* sensu stricto (s.s.), formerly known as “temperate lineage” (Nava et al. 2018). This provided new insights into the distribution and genetic divergence of the *Rh.* *sanguineus* group. During this redescription, two further lineages of *Rh. sanguineus* s.l. were redefined and described as independent species. Thus, the globally distributed "tropical lineage" was newly classified as *Rhipicephalus linnaei* and the "southeastern Europe lineage" distributed in Eastern Europe and the Middle East as *Rhipicephalus rutilus*. (Chitimia-Dobler et al. 2017; Laatamna et al. 2020, 2022; Chandra et al. 2020; Šlapeta et al. 2021, 2022, 2023; Hekimoğlu 2024).

*Rh. sanguineus* s.l. is closely associated with their main host, the dog (Hekimoğlu 2024). In endemic areas, these ticks are also found on a variety of other hosts, including humans (Millan et al. 2007; Dantas-Torres 2010; Mihalca et al. 2012a; Hornok et al. 2018). When introduced into non-endemic areas where outdoor conditions do not allow survival, these ticks are still able to establish, survive and reproduce indoors, also in insulated sheds or kennels (Walker et al. 2000; Dantas Torres et al. 2010). The combination of the permanent accessibility of dogs and suitable abiotic factors, like temperature, humidity, and availability of hiding places, can result in the rapid reproduction of the species. *Rh. sanguineus* s.l., especially their nymphs and larvae, are most frequently found on dogs' toes, ears, and axillary areas and therefore being overlooked by dog owners. According to Dantas-Torres et al. (2010), the stages of *Rh. sanguineus* s.l. prefer to feed on body areas with thin skin as their hypostome is quite short resulting in aggregated feeding of juvenile stages. After a blood meal, ticks leave their host and retreat to hidden places such as cracks and crevices, behind wallpapers, under carpets and even between floorboards, where they moult or lay up to 6000 eggs per fertilised female (Walker et al. 2000; Dantas-Torres 2010; Gray et al. 2013). Under favourable conditions, *Rh. linnaei* can reproduce up to four times per year, whereas *Rh. sanguineus* s.s. completes only one cycle (Dantas-Torres 2010; Gray et al. 2013; Labruna et al. 2017).

*Rh. sanguineus* s.l. can act as vector for several pathogens, harmful to both dogs and humans. Of particular importance in Europe is the vector competence for *Rickettsia* species, especially *R. conorii* and *R. massiliae*. Both belong to the tick-borne spotted fever group and are considered to be human pathogens (Gray et al. 2013) and cause only subclinical or mild symptoms in dogs (Levin et al. 2011). However, *R. massiliae* is more frequently detected in ticks in the Mediterranean region than *R. conorii* (Vitale et al. 2006; Parola et al. 2013; Monje et al. 2016). In addition, *Rh. sanguineus* s.l. is a vector for *Babesia vogeli*, *Hepatozoon canis* and *Ehrlichia canis* which cause prolonged and sometimes severe symptoms or lethal outcome in dogs (Menn et al. 2010; Hamel et al. 2011; Gray et al. 2013; Millan et al. 2016). However, the course of infection and severity of disease varies. While *B. vogeli* and *E. canis* are transmitted by tick bites, *H. canis* is an orally acquired pathogen that is transmitted by ingestion of an infected tick (Baneth et al. 2007; Waitz 2011; von Samson-Himmelstjerna 2019).

The first records of the introduction of *Rhipicephalus* species into northern Germany (Hamburg) were published in 1944 (Zumpt 1944). However, this study did not evaluate the travel history and import routes of dogs and ticks from abroad and did not provide a defined species identification, but as a result, *Rh. sanguineus* ticks were falsely considered an endemic tick species in Germany, even though there is no common distribution today (Zumpt 1944; Gothe and Hamel 1973). However, Gothe and Hamel (1973) pointed out that the development of imported *Rhipicephalus* species cannot be completed outdoors. Since then, the endemization of *Rhipicephalus* species has not been monitored in Germany, but the introduction of *Rh. sanguineus* has been repeatedly reported from the federal states of Baden-Württemberg, Hesse, Berlin, Hamburg, Bavaria and Lower Saxony (Zumpt 1944; Gothe and Hamel 1973; Hoffmann 1979, 1981; Centurier et al. 1979; Dongus et al. 1996), who mainly came from the USA, Morocco and the Mediterranean countries through tourism and military services (Gothe and Hamel 1973; Hoffmann 1981). Observations to date indicate that *Rh. sanguineus* is introduced by dogs that are imported from abroad or by travellers with their dogs, who apparently do not practice adequate tick prophylaxis (Dongus et al 1996; Menn et al. 2010, Abdullah 2016). Therefore, the knowledge about the frequency of introduction into Germany, survival and reproduction rates of *Rhipicephalus* species is limited, as the genus is not a notifiable pest in Germany. The present study intends to provide more information and to fill these knowledge gaps.

## Material & methods

### Citizen-science study

To investigate the pathways of tick importation focused on the genus *Rhipicephalus* into Germany and to document established tick infestations in houses, a citizen-science study was conducted. In 2018, a nationwide appeal was launched in Germany to send in ticks associated with travel or were collected in the context of a tick infestations in houses, especially *Rh. sanguineus* s.l. for genetic species identification and associated pathogens. This appeal has been renewed annually from 2019 to 2021. To give more information about the project as well as the tick species, a website was launched offering information on the requested ticks species, including images (https://hundezecken.uni-hohenheim.de/hundezecken 04 March 25 10:20). Another aim of this study was to raise awareness of the introduction of *Rh. sanguineus* s.l. and other species of the genus *Rhipicephalus* among professionals such as veterinarians, employees of animal shelters and pest controllers. It became apparent that veterinarians and pest controllers in Germany are often unfamiliar with *Rh. sanguineus* s.l. which are often introduced with dogs. The misidentification of *Rhipicephalus* species as endemic tick genera of Germany such as *Dermacentor* spp. or *Ixodes* spp., as mites or fleas is still very common and can lead to long-term *Rhipicephalus* infestations on dogs and in houses. Therefore, informative flyers and posters on the morphology and behaviour of *Rh. sanguineus* s.l. were distributed to a total of 226 institutions in Germany such as dog clubs, animal shelters, animal welfare societies, veterinary clinics, and local veterinarians as well as professional pest controllers to inform these relevant groups about the project. In addition, articles were published in specific journals named “Kleintiermedizin”, “Pest Control News” and “DpS” and a continuous media presence was maintained to raise awareness among interested readers and specialists in the respective fields about the introduction of ticks from abroad with a focus on *Rh. sanguineus* s.l. (Fachet and Mackenstedt 2020, 2021, 2024). The ticks sent in were subjected to molecular biological species identification and screening for associated pathogens transmitted by the ticks. The study was carried out between July 2018 and May 2024.

### Case documentation

For qualitative analysis of tick infestations of *Rh.* *sanguineus* s.l. or other *Rhipicephalus* species in Germany several parameters were included. Case documentation comprised the possible origin of tick infestations (including routes of travel or import), the intensity of tick infestation on the dog and inside of houses based on the number of ticks and different developmental stages, the possible exposure of other animals or humans, the tick prophylaxis used on dogs prior to tick infestation, and previous anti-tick treatments inside of buildings by the owner or professional pest controllers during the infestation in a non-endemic area of this tick.

The infestation density classifications “high”, “medium” and “single” include characteristics based on the number of ticks and the presence of developmental stages on dogs and in houses, of which at least one must be fulfilled for a case to be assigned to: High – At least two different developmental stages of ticks were collected in dwellings or on dogs. Ticks appeared in high numbers (> 100). People were not able to control and reduce the infestation by manual removal of the ticks and the administration of acaricides to dogs.

Medium – ticks of a single developmental stage were collected. Ticks appeared in lower numbers between 2 and 100 individuals. The infestation could be eradicated by the manual removal of ticks and the administration of acaricides to dogs.

Single—a single tick was detected on the dog, on its equipment, or inside a house.

The background of the tick import was assigned to three different categories named “travel”, “animal rescue” and “unknown”. The category "travel" includes all cases resulting from private travel activities with dogs, while the category "animal rescue" describes cases in which the dogs were either imported into Germany from abroad with self-organized campaigns, or the infested dogs originated from animal welfare organizations that import dogs from abroad, including animal shelters in Germany. It also includes the short-term stay of dogs from abroad at foster carers or families until adoption. The category "unknown" was selected if the background information was insufficient or the route of introduction was unclear.

### Preparation of ticks

Tick individuals of the genus *Rhipicephalus* sent to the University of Hohenheim were individually coded and sex as well as developmental stage were determined. Ticks were manually cut into pieces for genetic species identification and pathogen detection as described in chapters 2.4. and 2.5.

### Species identification of ticks

The species identification of sent-in ticks was carried out by genetic analysis. For molecular species identification, analysis of the 16S rRNA gene according to methods of Mangold et al. (1998) and Hauck et al. (2019) was performed. If less than 30 specimens were submitted per case, all ticks were identified to species level. If more than 50 specimens were submitted, at least 30 specimens were identified, with no more than 20% of the ticks per case being included. All ticks from cases categorized as “animal rescue”, all ticks were identified to species level, as there might have been more than one introduction event which dramatically increases the likelihood of detecting infestations involving more than one tick species. Some cases could only be evaluated from photographs. In those cases, tick identification from the submitted images was made by visual assessment of the ticks to genus level.

DNA of individual ticks was extracted using Sodium hydroxide (Werner et al. 2002; Hüttner et al. 2008; Bucher et al. 2021). 1/4th of each tick was incubated in 20 µl NaOH (0,02 M) for 10 min at 95 °C to extract the DNA. Conventional PCR to amplify the 16S rRNA gene was performed using Dreamtaq DNA Polymerase (Thermo Fisher Scientific Inc.) with 3 µl of DNA. Primers, ingredients and temperature profile can be found in Tables 1, 2, 3. Amplicons were purified using High Pure PCR Purification Kit (Roche Deutschland Holding GmbH, Mannheim, Germany) and sent for sequencing to Microsynth seqlab GmbH (Göttingen, Germany). Sequence analysis and editing was performed using GENtle V1.9.4 (Manske 2003). A phylogenetic tree was generated in MEGA V11.0.10 as Maximum likelihood tree, calculated with modeltype T92 + G (Tamura 3-parameter + discrete Gamma distribution) and a bootstrap support of 1000 replications (Tamura et al. 2021).

**Table 1:** Primer and Probe sequences and publication list of examined gene targets and corresponding references

Gene target	PCR system	References	Primer/probes (10 µM)	Primer sequences (5′ to 3′)
All species* Rickettsia spp.* glta	qPCR	Modified, Wölfel et al. (2008)	PanRick glta 2F	ATAGGACAACCGTTTATTT
			PanRick glta 2R	CAAACATCATATGCAGAAA
			PanRick 3 Taq	(FAM) CCTGATAATTCGTTAGATTTTACCG (**BHQ1**)
*Rickettsia spp.* OmpB	Conventional PCR	Roux and Raoult (2000)	OmpB 120–2113	CGATGCTAACGTAGGTTCTT
			OmpB 120–2988	CCGGCTATACCGCCTGTAGT
*Hepatozoon canis *18srRNA	qPCR		ILO F	GTCAGAGGTGAAATTCTTAGATTTGT
		Modified Hawkins et al. (2015)	ILO 9030	ATTTCTCTCAA**K**GT**S**CTGAAG
			ILO Taq 2	(FAM) AATCAAGAACGAAAGTTAGGGGAT (**BHQ1**)
*Babesia vogeli* hsp70	qPCR	Modified, Paulino et al. (2018)	BvqF	GCTGGTGACACCCACCTT
			BvqR	GGCACGCTTGTTGGTC
			BvqS	(FAM) CCTCCTCGTTGAGCACT **(BHQ1)**
*Ehrlichia canis* 16SrRNA	qPCR	Modified; Baneth et al. (2009)	EcqF	TATAGCCTCTGGCTATAGGAAATTGTTA
			EcqR	ACCATTTCTAATGGCTATTCCGTACTA
			EcqS	**(TAMRA)** TGGCAGACGGGTGAGTAATGCGTAGG **(BHQ2)**
Tick species identification 16SrRNA	Conventional PCR	Mangold et al. (1998)	16 s-1 F	CCGGTCTGAACTCAGATCAAGT
			16S R	GCTCAATGATTTTTTAAATTGCTGT

Modifications are prefixed in bold

**Table 2:** Mastermix compositions for 1 sample, left/ middle: ingredients used for conventional PCR and right: ingredients for qPCR–Biozym Blue Probe qPCR Kit Separate ROX and Biozym Probe qPCR Kit Separate ROX with the same compositions

Amplitaq reaction volume 50 µl	Conventional PCR in µl	Dreamtaq reaction volume 50 µl	Conventional PCR in µl	Biozym reaction volume 20 µl	qPCR in µl
Buffer II	5	Dream buffer	5	PCR Mix	10
MgCL_2_ (25 mM)	4	Primer forward (10 µM)	2	Primer forward (10 µM)	0.8
Primer forward (10 µM)	2.5	Primer reverse (10 µM)	2	Primer reverse (10 µM)	0.8
Primer reverse (10 µM)	2.5	dNTP´s (10 mM each)	1	Probe (10 µM)	0,4
dNTP´s (10 mM each)	1	Dream Taq (5 U/µl)	0,3	H_2_O	5
AmpliTaq (5 U/µl)	0.25	H_2_O	36,7		
H_2_O	29.75				
DNA	5	DNA	3	DNA	3

**Table 3:** Temperature profiles of performed PCR systems and references

Name	Author	Profiles									
*Babesia vogeli, Ehrlichia canis,*	Modified, Baneth et al. 2009; Paulino et al. 2018	Temp. (°C)	95	93	60						
		Time	2 min	20 s	60 s						
multiplex qPCR		cycles		45x							
*Hepatozoon* sp.		Temp. (°C)	94	94	58	72					
		Time	5 min	15 s	15 s	30 s					
qPCR		cycles		45x							
All species* Rickettsia* spp.	Wölfel et al. 2008	Temp. (°C)	95	95	55	60					
		Time	2 min	10 s	20 s	30 s					
qPCR		cycles		45x							
*Rickettsia* spp.	Modified, Roux and Raoult 2000	Temp. (°C)	95	95	50	68	68	4			
		Time	3 min	30 s	30 s	90 s	7 min	∞			
OmpB		cycles		40x							
Tick species identification	Mangold et al. 1998; Hauck et al. 2019	Temp. (°C)	95	95	47 + 0.3/2 cycles	72	95	50	72	72	4
		Time	3 min	30 s	30 s	45 s	30 s	30 s	45 s	7 min	∞
16SrRNA		cycles		7x			35x				

### Pathogen detection in ticks

For pathogen detection 3/4th of each tick specimen was transferred to a 2 ml safe lock reaction tube homogenized in 400 µl cell culture medium (Gibco Minimum Essential Medium with GlutaMAX, Thermo Fisher Scientific Inc.) with 3 steel beads (Pentaton GmbH AT—cleaning beads 18/8) in a mixer mill (MM400, Retsch GmbH) at 30 Hz for 30 min. Homogenates of 5 ticks (40 µl each) were pooled and DNA was extracted using the Maxwell 16 Tissue DNA Purification Kit from Promega for DNA isolation in a Maxwell 16 System (Promega Corporation, Promega GmbH) according to manufacturer’s protocol.

Those pools were tested for *Rickettsia* spp., *E. canis*, *B. vogeli*, and *H. canis* by qPCR using the Biozym Blue Probe qPCR Kit Separate ROX (*Rickettsia* spp.*, H. canis*) and Biozym Probe qPCR Kit Separate ROX (*E. canis, B. vogeli*) (Tables 1, 2, 3). Ticks from pools that were tested positive for one of the pathogens were subjected to individual testing. Ticks tested positive in an all-species *Rickettsia* qPCR (Wölfel et al. 2008) were further analyzed to species level via the OmpB Gene as described by Roux and Raoult (2000) using AmpliTaq™ DNA Polymerase with Buffer II (Applied Biosystems™) as shown in Tables 1, 2, 3. Amplicons were sequenced by Microsynth Seqlab GmbH and sequence analysis was performed in GENtle V1.9.4 (Manske 2003). Sequences obtained were then compared with existing entries in GenBank using NCBI BLAST. The *Rickettsia* species were considered identified if the sequence comparison showed a homology of 99.7% or higher (Fournier et al. 2003).

## Results

### Case reports

Between 2019 and 2024, 42 different cases of infestations with ticks of the genus *Rhipicephalus* were reported from all over Germany as well as Austria and Switzerland with one case each (Table 4: R1-R44). 19 cases were classified as “high”, 16 as “medium” and nine cases reported only a single tick detection. In eight out of 44 cases tick prophylaxis prior to vacation or dog importation was reported, including both spot-on treatments and systemic acaricides (spot on = 6; systemic = 1; combination = 1).

**Table 4:** Overview of tick infestations of *Rhipicephalus* in Germany and categorization of case reports resulting from the citizen-science study between 2019 and 2024

	Year	Country of tick origin	Region of tick origin	Infestation density	Category	Background	Tick prophylaxis	Treatment	Tick species	Localisation of case
R1	2019	Hungary	Polgar	High	Animal rescue	Dog import	Spot on		*Rh. sanguineus* s.s	Baden-Wuerttemberg
R2	2022	Portugal		High	Animal rescue	Dog import	Unknown		*Rhipicephalus* spp.	Germany
R3	2020	Romania		High	Animal rescue	Dog import	No		*Rhipicephalus* spp.	Hesse
R4	2022	Greece	Lesbos	High	Animal rescue	Dog import	No		*Rhipicephalus* spp.	Germany
R5	2021	Egypt		High	Animal rescue	Dog import	No		*Rhipicephalus* spp.	Rhineland Palatinate
R6	2023	unknown		High	Animal rescue	Dog import	Spot on + systemic		*Rhipicephalus* spp.	Germany
R7	2020	unknown		High	Animal rescue	Infested animal shelter	No		*Rh. sanguineus* s.s	Baden-Wuerttemberg
R8	2019	unknown		High	Animal rescue	Infested dog day care	No		*Rh. sanguineus* s.s	Baden-Wuerttemberg
R9	2021	Hungary		High	Animal rescue	Dog import	No	Professional	*Rhipicephalus* spp.	Austria
R10	2019	Spain	Cadiz	High	Animal rescue	Dog import	Spot on		*Rh. sanguineus* s.s	Bavaria
R11	2021	Croatia	Pag	High	Travel	Vacation	No		*Rh. sanguineus* s.s	Bavaria
R12	2019	Slovenia	Bovec	High	Travel	Vacation	No		*Rh. sanguineus* s.s	Baden-Wuerttemberg
R13	2019	unknown		High	Travel	Infested holiday house	No	Professional	*Rh. linnaei*	Baden-Wuerttemberg
R14	2022	Croatia		High	Travel	Vacation	No		*Rhipicephalus* spp.	Germany
R15	2019	unknown		High	Unknown	Unknown	Unknown	Professional	*Rhipicephalus* spp.	Baden-Wuerttemberg
R16	2021	unknown		High	Unknown	Unknown	Unknown	Professional	*Rhipicephalus* spp.	North Rhine-Westphalia
R17	2022	unknown		High	Unknown	Unknown	No		*Rhipicephalus* spp.	Rhineland Palatinate
R18	2019	unknown		High	Unknown	Moving into infested dwelling	Unknown		*Rhipicephalus* spp.	Bavaria
R19	2022	unknown		High	Unknown	Moving into infested dwelling	No		*Rhipicephalus* spp.	Bavaria
R20	2022	Egypt		Medium	Animal rescue	Dog import	No		*Rhipicephalus* spp.	Germany
R21	2021	Spain	Murcia	Medium	Animal rescue	Dog import	Unknown		*Rh. sanguineus* s.s	North Rhine-Westphalia
R22	2022	Spain		Medium	Animal rescue	Dog import	No		*Rhipicephalus* spp.	
R23	2019	Spain	Lanzarote	Medium	Animal rescue	Dog import	No		*Rhipicephalus* spp.	Switzerland
R24	2022	Bulgaria	Pasardschik	Medium	Animal rescue	Dog import	No		*Rhipicephalus* spp.	Hamburg
R25	2022	Netherland		Medium	Travel	Infested holiday house	Unknown		*Rhipicephalus* spp.	North Rhine-Westphalia
R26	2020	Italy	Liguria	Medium	Travel	Treated 1 of 2 dogs	Systemic		*Rhipicephalus* spp.	Bavaria
R27	2020	unknown		Medium	Travel	Infested holiday house	Unknown	Professional	*Rh. sanguineus* s.s	Schleswig–Holstein
R28	2023	Croatia		Medium	Travel	Vacation	Unknown	Professional	*Rhipicephalus* spp.	Germany
R29	2022	Italy	Sardinia	Medium	Travel	Vacation	No		*Rhipicephalus* spp.	Germany
R30	2022	Spain	Mallorca	Medium	Travel	Vacation	No		*Rhipicephalus spp.*	Germany
R31	2022	Greece	Thassos	Medium	Travel	Vacation	Spot on		*Rhipicephalus spp.*	Baden-Wuerttemberg
R32	2019	Italy	Fratte Rosa	Medium	Travel	Vacation	No		*Rhipicephalus spp.*	Bavaria
R33	2020	Croatia	Pula	Medium	Travel	Vacation	Unknown		*Rh. sanguineus* s.s	Bavaria
R34	2024	unknown		Medium	Unknown	Moving into infested dwelling	Unknown	Professional	*Rhipicephalus* spp.	Germany
R35	2023	unknown		Medium	Unknown	Moving into infested dwelling	No		*Rhipicephalus* spp.	Germany
R36	2020	unknown		Single	Animal rescue	Infested dog day care	No		*Rhipicephalus* spp.	North Rhine-Westphalia
R37	2020	Sri Lanka		Single	Animal rescue	Dog import	No		*Rh. haemaphysaloides*	Baden-Wuerttemberg
R38	2022	Greece		Single	Animal rescue	Dog import	spot on		*Rhipicephalus* spp.	Baden-Wuerttemberg
R39	2019	Spain	Valencia	Single	Travel	Vacation	spot on		*Rh. sanguineus* s.s	Rhineland Palatinate
R40	2019	Egypt		Single	Travel	Vacation	Unknown		*Rh. rutilus*	Hesse
R41	2020	Croatia	Šibenik	Single	Travel	Vacation	Unknown		*Rh. linnaei*	Lower Saxony
R42	2019	Croatia	Pag	Single	Travel	Vacation	No		*Rh. turanicus*	Saarland
R43	2021	France	Alsace	Single	Travel	Vacation	Spot on		*Rh. sanguineus* s.s	Baden-Wuerttemberg
R44	2023	unknown		Single	Unknown	Unknown	Unknown		*Rhipicephalus* spp.	Germany

The year of reporting, the origin of the ticks, the number of tick density, the categorization of cases, special background of tick importation, the choice of tick prophylaxis prior the infestation in Germany, identified tick species through molecular species identification, as well as special circumstances of the infestation were assessed. In the treatment column, a distinction is made between professional treatment of the house by a pest controller and self-controlled removal of the ticks by participants (empty slot)

Within the cases classified as “high”, participants reported either a spot-on treatment (2/19 Table 4: R1, R10) or a combination of spot-on treatment and a systemic acaricide (1/19 Table 4: R6) or no use of tick prophylaxis at all (12/19) while in four cases no information was available (4/19). Ten cases originated from an "animal rescue" (10/19) and four cases from a "travel" (4/19), while five cases could not be categorized due to a lack of information on tick import history and are referred to as “unknown” (5/19). The professional treatment by pest controllers were necessary in four cases (4/19 Table 4: R9, R13, R15, R16).

Of all cases that were classified as "medium" infestation, five cases originated from "animal rescue" (5/16), whereas nine cases were acquired during "travel" (9/16) while the reason for the infestation could not be identified in two cases (2/16 Table 4: R34, R35). Only two participants reported the use of tick prophylaxis prior the travel (2/16). In one case the participants stated the use of a systemic acaricide on one of two dogs (R26) and in another case, a spot-on treatment was used before the vacation (R31). Three infestations in houses were treated by a professional pest controller (3/16 Table 4: R27, R28, R34).

Nine participants reported a “single” tick introduction. Three of these cases were categorized as "animal rescue" (3/9) five cases within the classification "single" were travel related (5/9), and only one case could not be assigned due to missing information (1/9). Three participants reported that a spot-on treatment as tick prophylaxis had been used prior the dog was imported (3/9) and no pest controllers were involved.

The treatment of dogs against ticks in Germany during the infestation included newly administered acaricides or spot-on treatments as well as manual removal of ticks from the dog. The selected methods by participants to remove ticks from buildings included the application of commercially available acaricides, manual removal of ticks by visual inspection, treatment of exposed fabrics with cold or heat (freezing and drying) and masking tick-exposed areas with double-sided tape.

A total of 30 of the participants were able to provide sufficient information to determine the route of introduction and the country of origin of the ticks (30/44) (Table 4). There is no prominent country of origin within “high”, “medium” or “single”. Ticks were imported from Spain and Croatia with six cases each. From Italy, Greece and Egypt ticks were introduced with three independent introductions each, followed by Hungary with two introductions. One tick introduction each originated in Slovenia, Romania, Portugal, France, Bulgaria, Netherland, and Sri Lanka. In 14 cases, the country of origin of imported ticks could not be determined (14/44).

**Fig. 1:**
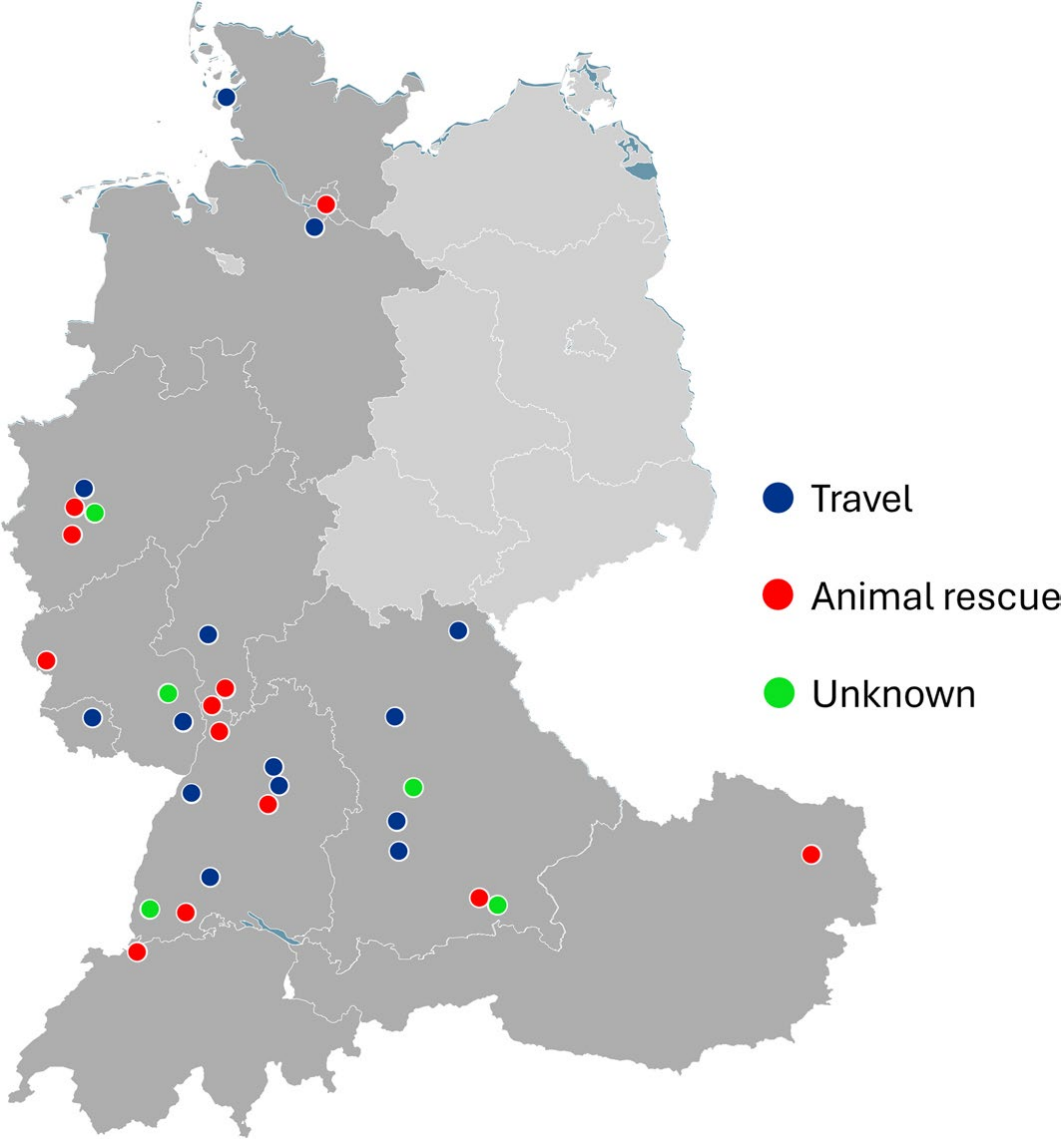
Cases of *Rhipicephalus* species infestations in houses or on dogs in Germany, Austria and Switzerland (32/44 cases) shown in the categories “Travel—blue”, “Animal rescue—red” and “Unknown—green” as import reason. 12 cases could not be localized due to missing information. Cases have been reported from the German states of Baden-Wuerttemberg, Bavaria, Hesse, Lower Saxony, North Rhine-Westphalia, Rhineland-Palatinate, Schleswig–Holstein and Hamburg, as well as from Vienna in Austria and the canton of Basel-Landschaft in Switzerland

During this project, cases were reported from Germany (42/44), Austria (1/44, R9) and Switzerland (1/44, R23), demonstrating the level of awareness that was achieved during this citizen-science study (Fig. 1). Although many dogs were imported from the same country or the study participants visited the same holiday country, no connection could be traced between the cases and each case was an independent introduction of the ticks.

### Tick species identification and phylogenetic analysis

In 17 of the 44 cases, ticks were available for confirmatory molecular species identification using the 16S rRNA gene (450 bp length) molecular data. As a result, one tick from Croatia was identified as *Rh. turanicus* (R42) and one tick from Sri Lanka was identified as *Rh. haemaphysaloides* (R37). The remaining ticks were identified as *Rh. rutilus* (R40), *Rh. linnaei (R13, R41) and Rh. sanguineus* s.s. (Table 4*)*.

**Fig. 2:**
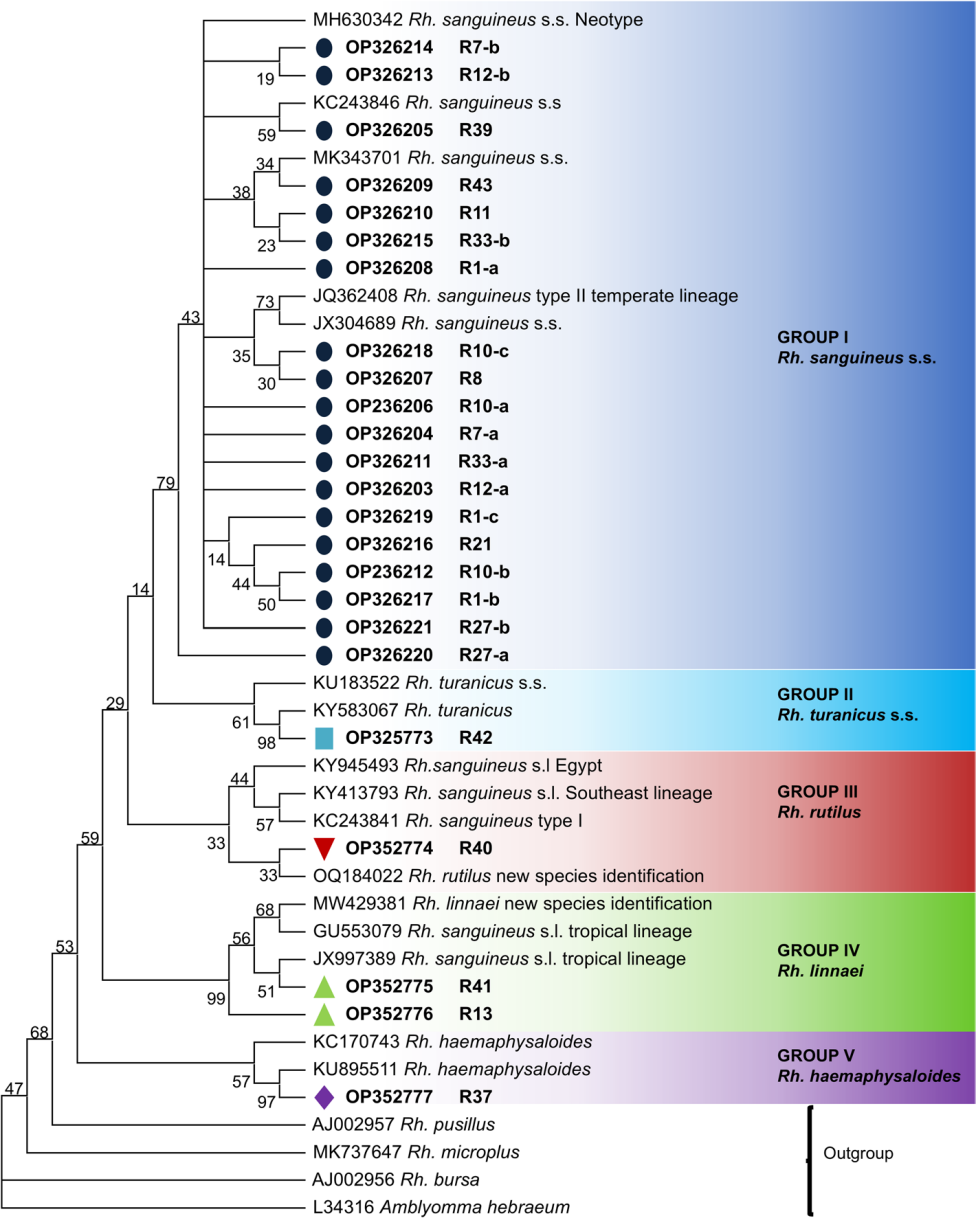
Phylogenetic Maximum likelihood tree based on 16S rRNA of *Rhipicephalus* spp. Included are the sequences from each case imported into Germany named according to the case nomenclature—marked with coloured circles, squares and triangles and prefixed in bold. The bootstrap value of each branch point is given next to the branches

In total, sequences from 164 tick individuals were analyzed for species identification. Identical sequences derived from the same case were only included once in the phylogenetic analysis and named according to the case nomenclature (R1–R44). If multiple genetic variations were detected per case, they were numbered consecutively in addition to the case nomenclature, e.g. R10-a to R10-c. All sequences were submitted to NCBI GenBank under the accession numbers OP326203–OP326221 and OP352773–OP352777 resulting in 24 sequences, which are shown in the phylogenetic tree (Fig. 2).

A total of 24 sequences could be obtained, clustering in five different groups formed by the following taxa: **Group I**- “*Rh. sanguineus* s. s.” including 19 sequences; **Group II**—“*Rh. turanicus* s. s*.*” including one sequence; **Group III** – “*Rh. rutilus”* including one sequence; **Group IV** – “*Rh. linnaei”* including two sequences and **Group V** – “*Rh. haemaphysaloides*” with one sequence.

**Fig. 3:**
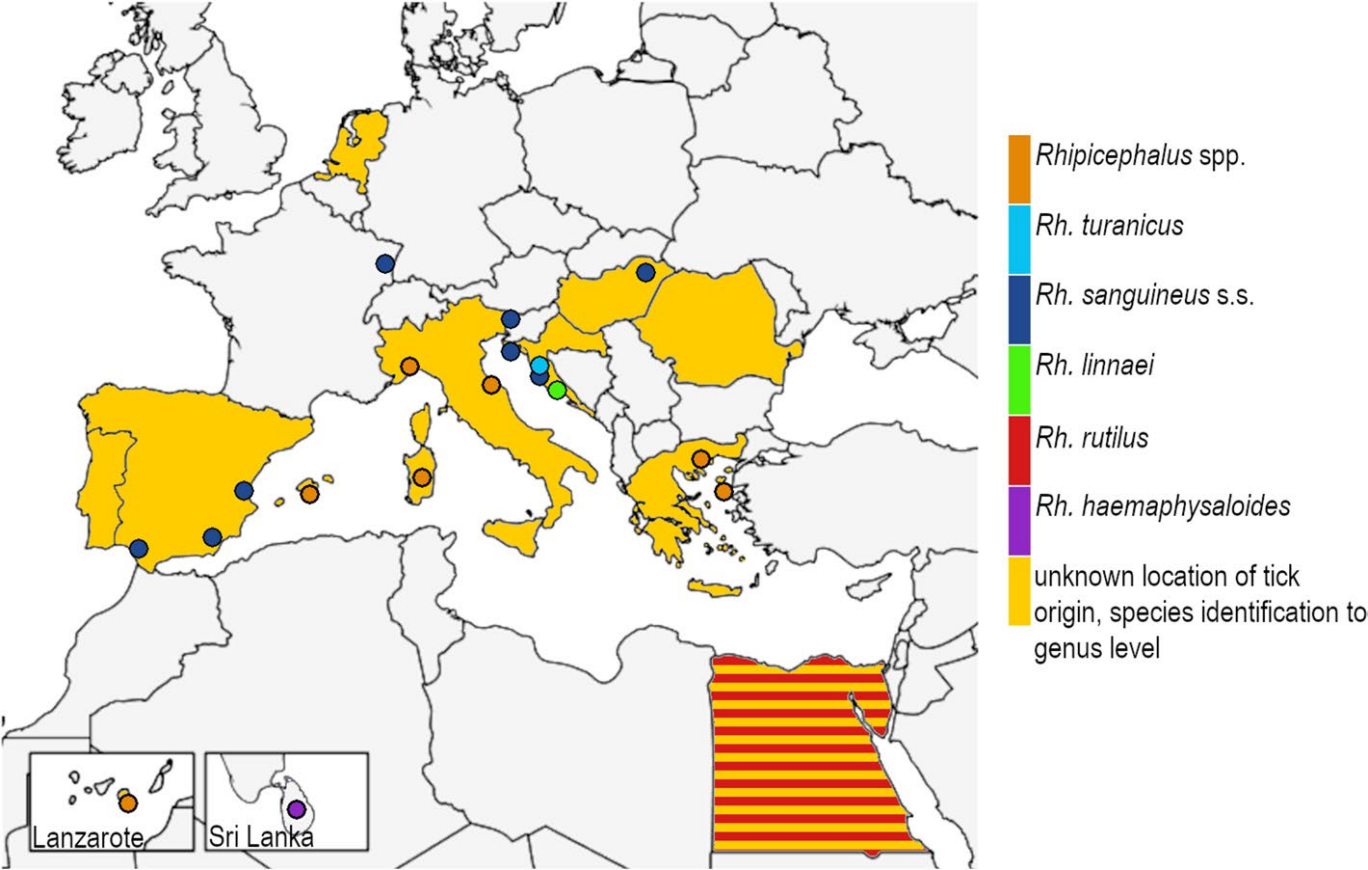
Origin of ticks reported by study participants between 2019 and 2024. Colors describe the associated tick species. Dots indicate exact location of tick origin; countries of origin without a known exact location are completely colored. Egypt represents the origin of an identified *Rh. rutilus* specimen as well as unidentifiable *Rhipicephalus* specimens, both of whose exact origin could not be clarified

The origin of the ticks is shown in Fig. 3. The distribution of the sent in *Rh. sanguineus* s.s. extends across western and central Europe from areas where they are known to be endemic, with one specimen originating from France, Alsace (dark blue in Fig. 3). The single *Rh. rutilus* could be traced back to Egypt and is marked in red. Both, *Rh. linnaei* and *Rh. turanicus* were introduced from Croatia (green and light blue, respectively). The only introduction from outside of the Mediterranean area and Europe was one single specimen of *Rh. haemaphysaloides* from Sri Lanka. Cases that could only be identified at genus level based on photographs are included in yellow-coloured countries or orange dots if the exact location was reported.

### Pathogen detection

A total of 780 tick individuals, received from 17 cases, were tested negative for *B. vogeli, E. canis* and *H. canis*. However, rickettsial DNA was detected in tick pools from six cases (R8, R10, R12, R13, R27, R39). Individual ticks from these pools were analyzed. A total of 50 ticks imported from either Slovenia (R12) or Spain (R10, R39) were tested positive for *Rickettsia *sp. by all species qPCR (Wölfel et al. 2008). The ticks from cases R8, R13 and R27 belonged to the cases whose origin was unknown (Table 5). The causative agents of 31 *Rickettsia* (cases R10, R27, R39) were identified as *R. massiliae* isolated from *Rh. sanguineus* s.s. in each case respectively. The origin of two cases was Spain (R10, R39), the third was unknown (R27). *Rickettsia* species identification of six infected ticks from cases R8, R12 and R13 was not possible due to low sequence quality. One *Rickettsia* sp. infection was detected in ticks of the species *Rh. linnaei* (R13) of unknown origin.

**Table 5:** Number of examined ticks and pathogen detections per case

Case	Identification of ticks	Country of origin	Examined ticks	*Rickettsia* spp. positive	*Rickettsia* species identification	Homologous sequences (NCBI)
R1	*Rh. sanguineus* s.s	Hungary	32	–	–	
R7	*Rh. sanguineus* s.s	unknown	61	–	–	
R8	*Rh. sanguineus* s.s	unknown	68	2	*Rickettsia sp.*	
R10	*Rh. sanguineus* s.s	Spain	298	18	*R. massiliae*	CP003319 97.78
R11	*Rh. sanguineus* s.s	Croatia	68	–	*–*	
R12	*Rh. sanguineus* s.s	Slovenia	191	3	*Rickettsia sp.*	
R13	*Rh. linnaei*	unknown	6	1	*Rickettsia sp.*	
R21	*Rh. sanguineus* s.s	Spain	6	–	–	
R27	*Rh. sanguineus* s.s	unknown	32	25	*R. massiliae*	CP003319 100%
R33	*Rh. sanguineus* s.s	Croatia	11	–	–	
R36	*Rhipicephalus* sp.	unknown	1	–	–	
R37	*Rh. haemaphysaloides*	Sri Lanka	1	–	–	
R39	*Rh. sanguineus* s.s	Spain	1	1	*R. massiliae*	CP000683 99.88%
R40	*Rh. rutilus*	Egypt	1	–	–	
R41	*Rh. linnaei*	Croatia	1	–	–	
R42	*Rh. turanicus* s.s	Croatia	1	–	–	
R43	*Rh. sanguineus* s.s	France	1	–	–	
Summary			780	50		

## Discussion

Members of the tick genus *Rhipicephalus* are not considered to be endemic in Germany. However, sightings and even infestations of houses are occasionally reported even in these latitudes. In the present study, 44 such incidents were investigated as a part of a citizen-science study to determine the origin, import routes, and possible pathogens carried by these ticks.

### Case reports

In 31 of the 44 *Rhipicephalus* cases examined, the country of origin of the ticks could be identified, with European countries being the most common. The number of cases categorized as "animal rescue" (18/44), which means the active import of infested dogs to Germany, included ticks from mainly European countries (Europe = 11, Africa = 2, Asia = 1, unknown = 4) as well as in the category “travel” (Europe = 15, Africa = 1, unknown = 2). This coincides with the most commonly introduced species *Rh. sanguineus* s.s., which is endemic to European countries. The increasing number of cases in Germany has highlighted the potential risk of *Rh. sanguineus* s.l. infestations in densely populated urban areas such as big cities. Given the adaptation of these ticks to urban environments (Gray et al. 2013), it is important to determine whether big cities are at a higher risk of rapid tick spreading due to dense colonization and a large number of dogs in a confined space. It is possible that transmission may occur between dogs through close contact between the animals, shared resting areas or shared housing. This could result in the spread of ticks from dogs to new indoor environments, such as dog day care centers, overnight stays, and so forth. Furthermore, the potential for ticks to migrate from one apartment to the next exists if dogs residing in the same building. Despite the high number of cases and the considerable number of tick individuals involved, there has been no evidence of transmission from dog to dog or between neighboring houses and apartments. Thus far, the ticks have been identified and eradicated in a timely manner, either through direct treatment with acaricides or indirect treatment of the dog with systemic acaricides. Those who have experienced an infestation with *Rh. sanguineus* s.l. have reported a notable increase in their level of alertness. This resulted in the immediate elimination of all host-seeking ticks and the limitation of contact with other dogs. Nevertheless, it can be postulated that *Rh. sanguineus* s.l. is capable of surviving and multiplying indoors for several years as long as an adequate host is available (Prosl and Kutzer 1986, Dantas-Torres et al. 2010, Gray et al. 2013). It has been suggested that frequent introductions may lead to the formation of autochthonous foci that exhibit epizootic spread (Hoffmann 1981). In Germany, it is currently unlikely that *Rh. sanguineus* s.l. will spread in the environment independently of dogs. As well, the mean annual temperatures are not sufficient to allow the development or survival of *Rh. sanguineus* s.l. or other imported *Rhipicephalus* species outdoors. Furthermore, the climate precludes survival beyond the winter or reproduction in outdoor areas (Hauck 2019, Labruna et al. 2017), despite the availability of suitable hosts in the fields such as the yellow-necked field mouse (*Apodemus flavicollis*), *Vulpes vulpes* (red fox) and *Meles meles* (European badger) (Millan et al. 2007; Gabrielli et al. 2010; Mihalca et al. 2012a, b; Umur et al. 2019).

#### Lack of tick prophylaxis

It appeared to be the critical matter of whether tick prophylaxis was carried out before the dog was imported or before the journey started. In 31 cases information was available on the use or the lack of tick prophylaxis, therefore only these cases can be discussed. In 23 of these 31 cases, no prophylactic measures were taken to prevent tick infestations resulting in a medium or high, long-lasting infestation in 20 cases (high = 12; medium = 8; single = 3). In summary, the majority of tick infestations were based on missing or insufficient tick prophylaxis.

In Germany acaricides for external use are available from vets in various forms of application such as spot-on formulations, shampoos, sprays, powders, and impregnated collars. The active compound is either fipronil, amitraz or pyrethroids, which are very effective against endemic tick species like *Ixodes* spp. and *Dermacentor* spp, as well as for *Rh. sanguineus* s.l. if used according to the manufacturer's instructions (Dantas-Torres 2008; Constantin et al. 2012; Dautel et al. 2013; Pages et al. 2014). Repellent products with natural ingredients commercially available in Germany are based on geraniol and/or essential oils of the tea tree, lemons, lavender or eucalyptus and similar organic ingredients more or less effective as tick repellent (Štefanidesová et al. 2017). However, spot on products can lose their effectiveness if used incorrectly, including bathing the dog, using after the expiry date, or not renewing them after the time specified by the manufacturer (Pfister and Armstrong 2016).

Only eight participants used repellents and/or acaricides as tick prophylaxis, including one systemic acaricide treatment (R26), six spot-on treatments (R1, R10, R31, R38, R39, R43), and in one case a combination of both (SI). It is not known which products were used and therefore it is unclear whether the spot-on products had a repellent effect or if they had an additional acaricidal effect. Spot-on products, either chemical-based or organic with essential oils, are not a sufficient way to prevent infestations with *Rh. sanguineus* s.l. as they are capable of surviving the repellent's period of action without a blood meal. This was shown in three travel associated cases (R31, R39, R43), where spot-on products were used with only a repellent effect. The study participants were aware of ectoparasites at their travel destination, so they decided to use a spot-on product with repellent effect as tick prophylaxis. Although a tick prophylaxis was used *Rh. sanguineus* s.s. individuals were imported into Germany, presumably with the dog bed and the dogs' harness leading to massive tick infestation with several hundred tick individuals. The use of repellents to prevent *Rh. sanguineus* s.l. from being introduced seems to be as limited as essential oils. So in case (R26) in the category “travel”, a systemic treatment was chosen for one dog, whereas the second dog was treated with essential oils as tick prevention leading to an infestation classified as “medium”. For veterinary reasons it was not possible to treat both dogs, so coconut and caraway oil was applied on the fur of the second dog. While the dog treated with the systemic acaricide was administered showed no tick infestation, the dog treated with the oils was infested with ticks of the genus *Rhipicephalus*.

Another three cases resulted from the import of an infested dog from abroad by an animal welfare organization despite the use of a tick prophylaxis. Two cases indicated spot-on products (R1, R10) and one case indicated the combination of a systemic acaricide and a spot-on (R6). The application of tick prophylaxis was carried out abroad by members of the animal welfare organization and was therefore not the responsibility of the participants. Accordingly, it cannot be ensured which acaricides were used or whether the acaricides were applied correctly. However, all three cases lead to tick infestations that were classified as “high”.

Effective protection against tick-associated pathogens can only be achieved by appropriate tick prophylaxis or/and avoidance of tick-infested areas. As the latter is often not feasible, it is recommended to apply systemic acaricides or spot-on medications with a combined repellent and killing effect against ticks on dogs prior to travelling to endemic distribution areas of *Rhipicephalus* species (Waitz 2011; Halos et al. 2012). Nevertheless, when using spot-on products, the dog must also be visually inspected for ticks.

#### “Travel”- related cases

18 of 44 cases are related to travel, therefore originating from dog owners travelling abroad with their dogs. Common to all is the total lack of acaricides and repellents used (8/18) or the incorrect application of such products (4/18) as described above. Only in three cases the participants travelled to areas in Germany and the Netherlands where *Rh. sanguineus* s.l. is not endemic and tick prophylaxis against native ticks such as *Ixodes* spp. and *Dermacentor* spp. was considered optional as the active phase of these endemic tick species and a careful search for ticks on dogs can make tick prophylaxis unnecessary. The situation is similar in these two cases (R13, R27), where holiday houses as frequently visited destination for dog owners were infested with *Rh. sanguineus* s.l. It is not possible to fully reconstruct the origin of the ticks before they infested the holiday houses, but it can be strongly assumed that former tenants and their dogs carried them from endemic areas. The case of R13 resulted in the transfer of *Rh. linnaei* to Germany, where the ticks multiplied unnoticed for weeks and then appeared in their thousands. The tick infestation was first recognized when larvae crawled out of the floorboards. The infestation was limited to three rooms where the dog preferred to stay. After several attempts to control the infestation with commercial acaricides, and after more and more ticks appeared over weeks (several hundred individuals in all developmental stages), a pest controller was involved, who stopped the infestation by fogging of cypermethrin. In case of R27, *Rh. sanguineus* s.s. were discovered on site by tourists and eradicated by the owners of the holiday home before they were transferred to Germany. These cases demonstrate the possibility of transferring *Rh. sanguineus* s.l. within non endemic areas and clearly show that the spread to non-endemic areas occurs and therefore tick prophylaxis is recommended regardless of the holiday destination.

#### “Animal rescue” related cases

The same number of cases are related to the category “animal rescue” as to the category “travel” (n = 18). Importing dogs into Germany from countries where *Rh. sanguineus* s.l. is endemic can also lead to a potentially high tick burden in homes. To date, there are more than 64 animal welfare organizations listed in the “German Register of Associations” that explicitly promote dog imports from abroad on their websites (Vereinsregister Deutschland 2023). The number of stray dogs in these countries is very high and many of them are caught and transferred to euthanasia stations (Heesen and Wendland 2019; Norman et al. 2020; Vereinsregister Deutschland 2023). The German Animal Welfare Association (DAW) has identified a specific need to provide assistance to rescue organizations for dogs in Greece, Italy, Croatia, Spain, Turkey, Hungary and Romania. (Deutscher Tierschutzbund e.V. 2022). The countries in question represent a portion of the natural range of *Rh. sanguineus* s.l. (Walker et al. 2000; Estrada-Peña et al. 2017), emphasizing the importance of tick prophylaxis before importing dogs and the awareness of animal welfare organizations, to prevent the introduction of non-endemic ticks.

The results of the case analyses are based almost entirely on the information provided by the participants. Therefore, it must be emphasized that the following discussion material contains personal views of the study participants. In addition, the present citizen-science study only included people who dealt with these ticks or are currently experiencing problems with ticks of the genus *Rhipicephalus*. So, the examples described below reflect isolated experiences that are not representative for all dog imports organized by animal welfare organizations and private imports of dogs from abroad.

Most cases categorized as "animal rescues" (10/18) are classified as highly infested. These dog imports, except for cases R7, R8 and R36, involved animal welfare organizations from abroad with direct handover of the dog to the new dog owners. Neither temporary foster homes nor animal shelters were included as a stopover until adoption. Although the animals were infested with ticks upon their arrival, it was the responsibility of the new owners to provide the initial veterinary treatment. These Dogs were treated by a veterinarian within 48 h after arrival. This, however, is enough time for various ectoparasites such as ticks and fleas to be introduced into private homes. Participants reported that the involved rescue organizations and private animal welfare associations were not able to pay more attention to the veterinary care of the dogs, including the treatment of ectoparasites, due to the lack of funds. The stated aim of these organizations is to rescue dogs from killing centers and poor living conditions in their countries of origin. The focus is on diagnosing possible diseases rather than treating ectoparasites. Seven participants stated that they were not adequately prepared for the dog´s condition focused on ectoparasites by the animal welfare organization and were surprised by the high tick burden on the dog itself (R1, R5, R9, R10, R21, R23, R24). The participants of the cases R1 and R10 were informed of the spot-on treatment prior to importation. Nevertheless, the dogs were “shockingly” covered with *Rh. sanguineus* s.s. individuals. In contrast, the participants of cases R2, R3 and R37 were informed in advance that the imported dogs had not received any veterinary treatment and expected a high tick burden. 10 out of 18 cases categorized as “animal rescue” developed into severe infestations. The cases R20, R21, R22, R23 and R24 which were classified as “medium” infestation rate, reported several juvenile ticks between the dog´s toes and/or on the muzzle, whereas the participants of R37 and R38 only found a single tick attached to the dog. The tick individuals were recognized quickly and eliminated immediately.

Only the cases R7, R8 and R36 did not emerge from the import of a dog from abroad, but from a short stay of dogs in a dog day care center (R8, R36) and the adoption of a dog out of an animal shelter in Germany that had a connection with the import of dogs from abroad (R7). The case R8 describes an infested dog day care center, which temporarily takes in dogs from people in need, including dogs from abroad. The origin of infestation regarding importation event or country of origin could not be determined but tick individuals were identified as *Rh. sanguineus* sensu stricto. However, the environment of the partially permanent dogs that are not treated with acaricides provided a perfect environment for *Rh. sanguineus* s.s. to multiply. Dogs placed in this day care centre were also at risk of a tick bite or spreading tick individuals. After such an outbreak, the dog day care center was closed by the owners. In contrast, case R7 describes an animal shelter that provides a temporary home for dogs from abroad until they are adopted. In this case, the private dogs of the shelter managers who accompany their owners to the shelter every day spread the infestation from the shelter to the private home. The participants´ dog from R36 visited a dog day care center where an infested dog that has recently been imported from abroad had stayed temporarily, but only a single tick was transferred.

Non-endemic tick species such as *Rh. sanguineus* s.l. pose a high risk to those who import dogs from abroad or offer them a temporary home. Particularly in places where many dogs are kept in restricted areas such as in public institutions (animal shelters) and at dog service providers (dog day care centers), the conditions are favourable for *Rh. sanguineus* s.l. to multiply or spread rapidly due to the high availability of hosts and the fluctuating change of these. Additionally, non-endemic ticks such as *Rh. sanguineus* s.l. expose humans like shelter employees or dog owners and of course other dogs to transmissible pathogens (Gray et al. 2013).

#### Category “unknown”

In total eight cases had to be categorized as “unknown”. Due to the incomplete nature of the information provided by email, it is not possible to make any further precise interpretations in three cases (R16, R17, R44). However, R15 could represent an apparently autochthonous case from southern Germany. In 2017, a severe infestation of ticks was reported in the yard of a car tire reconditioner with several hundreds of thousands of individuals (personal observation). In this case, the ticks were detected on piles of tires, but also everywhere else on the premises and in various sheds. The two guard dogs of the fenced in the yard did not leave the premises, except for the meadow a few meters away, so the infestation was limited to the property. According to the owner, there was no travel history of the dogs, nor did they knowingly come into contact with other dogs. The exact circumstances of the tick establishment could not be clarified but the high number of ticks indicated a long-term infestation. As the property was situated at the periphery of a village, it is possible that an infested dog from the village may have spread ticks around the perimeter of the property, making an autochthonous infestation possible. However, a link with an endemic *Rh. sanguineus* s.l. distribution area could also be made, as the main business is the regular import of used tires from Italy, which were stored on the site. The transport of *Rh. sanguineus* s.l. with car tires has not yet been described, but the preference of *Rh. sanguineus* s.l. to hide in cracks and crevices and to lay eggs or molt there also makes this route of tick import into Germany possible. Hoffmann (1981) already reported that *Rh. sanguineus* individuals arrived in Germany via a sheepskin rug from Morocco and a shopping basket from Portugal, supporting the idea of tick importation via tyres. At least four cases show that a *Rhipicephalus* spp. infestation can be present even without any dog ownership (R18, R19, R34, R35). The study participants moved into accommodations that were already infested. As there is no information on the previous tenants/owners, these cases could not be traced any further. However, it can be assumed that they were dog owners.

### Species identification and phylogenetic analyses of ticks

*Rh. sanguineus* s.s. was the most frequently imported tick species to Germany identified in 11 of the 17 cases analyzed molecularly. Chitimia-Dobler et. al. (2019) showed that *Rh. sanguineus* s.s. is endemic from the Canary Islands (Lanzarote) to Slovenia. This *Rhipicephalus* species is the most widespread taxon of *Rh. sanguineus* s.l. in Europe, which is also reflected by the results of this study. Also, imported *Rh. rutilus* and *Rh. turanicus* s.s. could be traced back to known endemic areas like Egypt and Croatia (Dantas-Torres et al. 2013; Chitimia-Dobler et al. 2017; Hornok et al. 2017; Nava et al. 2018).

As an unusual origin of ticks, participants in case R43 reported that they were hiking in Alsace, France, when their dog presumably acquired the ticks, later identified as *Rh. sanguineus* sensu stricto. It is an indication of a naturally free-living tick population if dogs get infested while walking. Contrary to older distribution maps by Walker et al. (2000) and Estrada-Peña et al. (2017), the eastern part of France has more recently been confirmed as an area with at least two established populations of *Rh. sanguineus* s.l., as shown by the current ECDC map of *Rh. sanguineus* reports, based on data from VectorNet project (ECDC 2023). In recent decades, the introduction of *Rh. sanguineus* s.l. into houses or environments in non-endemic areas has been a common occurrence in many areas in Europe, demonstrating the anthropogenic impact on the spread of ticks (Centurier et al. 1979; Szymanski 1979; Cerný 1989; Hornok et al. 2020; Buczek and Buczek 2021; Didyk et al. 2022). Even, if these introductions are mostly temporary, an established *Rh. sanguineus* s.l. population on wild ruminants has already been observed in East Romania and a northward spread of this tick was predicted for the future (Mihalca et al. 2012b; Alkishe et al. 2020). So *Rh. sanguineus* s.l. can also colonize and establish in new environments outside. Stable populations of *Rh. sanguineus* s.l. inside of dwellings are possible, but year-round survival outside of buildings in areas with colder climates (< 20 °C annual temperature) has not been published yet (Estrada-Peña et al. 2017; Walker et al. 2000). Therefore, it is possible, although still unlikely, that there is a temporary population of *Rh. sanguineus* s.s. surviving outdoors in Alsace, France. The presence of this possible population needs to be addressed and closely monitored, especially as the nearby Upper Rhine region, the warmest area in Germany, is an ideal establishment area and ticks can spread from France into Germany.

As a non-European representative species of *Rh. sanguineus* s.l., *Rh. linnaei* is distributed in areas between latitudes 35°N and 20°S (Šlapeta et al. 2021). Two independent introductions have been identified, but the source of introduction remains unclear in both cases. The case of R13 demonstrates nicely how difficult it is to trace back the origin of the ticks. Six individuals were sent in for molecular species identification, all identified as *Rh. linnaei* with identical sequences (OP352776). Neither the travel history of the dog nor other contacts abroad were reported. The most probable location where the ticks could have been picked up was a holiday home in northern Germany where participants of R13 stayed in 2019. The owners of this holiday home stated a high level of rentals in 2019 by dog owners, each of whom could have introduced *Rh. linnaei* specimens, but the holiday home itself showed no infestation. The tenants' countries of origin were not named. Therefore, the origin of these *Rh. linnaei* specimens could not be determined more precisely.

In contrast, the participants of case R41 initially stated that they had found the tick after hiking in Germany in Lower Saxony. In addition to many *Dermacentor* spp. ticks, the participant sent in a tick, later identified as a female *Rh. linnaei*. As reported, only a vacation to Pakoštane, Croatia, with daily trips to Zadar, Šibenik and the Krka National Park two years earlier was recognized as a stay abroad. Even if Croatia, especially in the areas around Pula, Zagreb, Zadar, Šibenik and Rovinj, is representing an endemic distribution area for *Rh. sanguineus* s.s., *Rh. turanicus* and *Rh. bursa* (Hornok et al. 2017; Chitimia-Dobler et al. 2019; Krčmar et al. 2022) there is still no evidence of an established population of *Rh. linnaei*. The two years between finding the tick and travelling to Croatia is also impossible to go unnoticed. Therefore, and also because of the long time between the holiday and the tick find, Croatia was excluded and the origin of this single specimen of *Rh. linnaei* remains unclear.

*Rhipicephalus haemaphysaloides,* which was also identified in the present study*,* is a species endemic to large parts of Asia (Walker et al. 2000) which was imported to Germany as a single specimen alongside a stray dog directly from Sri Lanka (R37). *Rh. haemaphysaloides* is endemic in Sri Lanka and can be found on stray dogs (Bandaranayaka et al. 2022).

### Pathogen detection

The results of the molecular survey of pathogens detected *Rickettsia* sp. in 50 of 780 examined ticks from six cases (R10, R12, R8, R27, R13, R39) with no evidence of *H. canis*, *E. canis* and *B. vogeli*. Specifically, *R. massiliae* was detected in 44 out of 50 *Rickettsia* sp. positive ticks (n = 50/780), while in six ticks *Rickettsia* species could not be identified. The remaining 730 ticks out of 11 cases were tested negative for *Rickettsia* spp. 88% of all *R. massiliae* were detected in *Rh. sanguineus* s.s.

The 44 detected isolates were most closely related to the *R. massiliae* strain AZT80 (CP003319) from Arizona with 99.75–100% (R27) and *R. massiliae* MTU5 (CP000683) with 99.88% (R39) similarity compared to sequences from GenBank. *R. massiliae* strain AZT80 is according to Eremeeva et al. (2006) 100% identical (based on 16S rRNA, 905 bp) to the strain Bar29 from Catalonia, Spain, a representative *R. massiliae* strain in Europe (Beati et al. 1996; Bernasconi et al. 2002; Marié et al. 2012; Parola et al. 2013; Mesquita et al. 2022). Case R10 achieved only 97.78% concordance with the *R. massiliae* strain AZT80 when compared with published sequences in NCBI and therefore cannot be assigned to the same species according to the definition of Fournier et al. (2003). In the absence of further genetic data, the species cannot be determined more precisely. *R. massiliae* MTU5 was firstly isolated from a *Rh. turanicus* collected on horses in Camargue, France (Blanc et al. 2007). The exact distribution of this genotype is not yet known, but the origin of the tick is Spain, Valencia. *R. massiliae* is part of the spotted fever group and considered as human pathogen (Barlett et al. 2022).

One of six specimens of *Rh. linnaei* (R13) was *Rickettsia* positive. However, the identification of the *Rickettsia* species could not be achieved, although multiple methods were performed. Although *Rickettsia* sp. was detected by qPCR, neither the amplification of OmpB (Roux and Raoult 2000), nor glta (Nilsson et al. 1999; Hartelt 2004), or OmpA (Regnery et al 1991; Pluta 2011) was successful. *Rhipicephalus linnaei* is a competent vector for *R. rickettsii*, *R. conorii caspia*, *R. conorii conorii*, *R. conorii israelensis*, *R. africae* and *Rickettsia* sp. strain Atlantic Rainforest (Dantas-Torres 2010; Parola et al. 2013; Monje et al. 2016; Slapeta et al. 2021). However, the possibility that the *Rickettsia* species belongs to the spotted fever group, rather than to the endosymbiotic *Rickettsia*-like species, which are also detected by the chosen qPCR method, cannot be ruled out (Ahantarig et al. 2013; Salomon et al. 2022). This would explain the unsuccessful attempts at genotyping. In addition to transmissible pathogens of the genus *Rickettsia*, *Rh. linnaei* is a known vector of *E. canis (*Labruna et al. 2017), which was absent in any tick during this study (R13 n = 6, R41 n = 1).

Case R27 is notable for the apparent high prevalence of *Rickettsia* in the examined *Rh. sanguineus* s.s. tested in this case. This case describes an infested holiday house in the northern Germany with frequently changing tenants, including dog owners. The infestation was handled by a pest controller. Therefore, only 32 adult ticks were sent in and could be analyzed of which 25 individuals were tested positive for *R. massiliae*. As *Rickettsia massiliae* is transmitted both transovarially and persists transstadially (Matsumoto et al. 2005), it can be speculated that either infected engorged females that lay eggs or a large number of infected *Rhipicephalus* nymphs that molt were brought into the apartment by previous visitors. The newly laid eggs would form the basis of a generation of infected ticks, which would settle in the house and be able to molt due to the frequent availability of hosts, such as travelers with dogs. This case illustrates how holiday homes used by many guests can contribute to the spread of *Rhipicephalus*. It also shows that the transfer of pathogens to the next developmental stage or generation increases the risk of humans and dogs coming into contact with these pathogens.

### Conclusion

Travelling with dogs, as well as importing dogs from Southern and Eastern European countries and even more distant countries such as Sri Lanka to Germany poses the risk of introducing ticks and their associated pathogens. The actual number of introductions of *Rhipicephalus* spp. either by dogs or in imported goods is certainly much higher than the number of cases documented in this citizen-science study, possibly because *Rh. sanguineus* s.l. are often misidentified by dog owners as a tick species endemic to Germany and therefore discarded without being recognized.

Nevertheless, there were a total of 44 cases between 2019 and 2024. Most of these cases were characterized by the number of ticks found either on the dog or already in the house. Although the cases could be eliminated, the participants of this study agreed with the statement that a tick infestation inside of houses is perceived as a psychological burden. Similarly, the financial burden must be taken into account, be it for the veterinary treatment of dogs, or the professional removal of ticks in houses by pest controllers. More than half of the cases in this study had in common that no tick prophylaxis was applied before the dog arrived at Germany, and eight cases show the insufficient use of acaricides that led to an import of *Rhipicephalus* species. However, the results also showed that the spread of an existing *Rh. sanguineus* s.l. infestation within Germany is possible. As ticks of *Rh. sanguineus* s.l. are vectors for various pathogens, including Rickettsiales such as *E. canis* and *R. conorii*, only the less infectious human pathogen *R. massiliae* could be detected in 44 introduced tick individuals. Nevertheless, these infected ticks represent the possibility of pathogens that can be carried by introduced ticks.

In 35 cases more than one tick of *Rh. sanguineus* s.l. have been imported into Germany and were able to survive and reproduce indoors, leading to sometimes severe infestations. Therefore, it is important to prevent the introduction of non-endemic tick species into Germany, especially by pets, as it is, for example, already established in UK (Fooks and Johnson 2015; Abdullah et al 2016; Norman et al 2020). Even if this extensive control cannot be realised in Germany in the same way as in the UK, general awareness among dog owners, animal welfare organizations, veterinarians and border/air control is essential to prevent tick introductions.

## Supplementary Information

Below is the link to the electronic supplementary material.

Supplementary file1 (PDF 191 KB)

## Data Availability

The datasets analyzed and included as results during the current study are available from the corresponding author on reasonable request.
