# Supplementary material for: Unwanted souvenirs—import routes and pathogen detection of the non-endemic tick Rhipicephalus sanguineus s.l. in Germany
Source: Exp Appl Acarol. 2025 Mar 11;94(3):42. doi: 10.1007/s10493-025-01010-0 (PMC11897088; doi:10.1007/s10493-025-01010-0)
Supplement: Supplementary file 1 — Supplementary file1 (PDF 191 KB) [file 10493_2025_1010_MOESM1_ESM.pdf]

Attachment

Table 1: Reference sequences used for phylogenetic analyses included from Genbank in.

**Accession Nr. NCBI**	**genetic identification**	**reference**	**comments**
MK343701	*Rh. sanguineus s.s.*	Chitimia-Dobler et al. 2019	
KC243846	*Rh. sanguineus s.s.*	Dantas-Torres et al. 2013	referred as sensu stricto by Chitimia-Dobler, et. al. 2017
JX304689	*Rh. sanguineus s.s.*	René- Martellet et al. 2015	
MH630342	*Rh. sanguineus s.s.* Neotype	Nava et al. 2018	designated neotype
JQ362408	*Rh. sanguineus s.l.* type II temperate lineage	Rene et al. 2012	assigned to sensu stricto
KY413793	*Rh. sanguineus s.l.* southeast lineage	Chitimia-Dobler et al. 2017	referred as southeast lineage
KC243841	*Rh. sanguineus s.l.* type I	Dantas-Torres et al. 2013	referred as southeast lineage by Chitimia Dobler, et. al. 2017
KY945493	*Rh. sanguineus s.l.* Egypt	Senbill et al. 2022	
OQ184022	*Rh. rutilus* new species identification	Slapeta et al. 2023	
GU553079	*Rh. sanguineus s.l.* tropical lineage	Moares-Filho et al. 2011	
MW429381	*Rh. linnaei* new species identification	Slapeta et al. 2021	
JX997389	*Rh. sanguineus s.l.* tropical lineage	Sanches et al. 2012	
KU183522	*Rh. turanicus* s.s.	Xu et al. 2015	
KY583067	*Rh. turanicus*	Li et al. 2017	
KC170743	*Rh. haemaphysaloides*	Sumrandee et al. 2012	
KU895511	*Rh. haemaphysaloides*	Nimisha et al. 2019	
AJ002957	*Rh. pusillus*	Marquez et al. 1997	outgroup
AJ002956	*Rh. bursa*	Marquez et al. 1997	outgroup
MK737647	*Rh. microplus*	Abdullah et al. 2019	outgroup
L34316	*Amblyomma hebraeum*	Black et al. 1994	outgroup
